# Medication-related visits in a pediatric emergency department: an 8-years retrospective analysis

**DOI:** 10.1186/s13052-017-0375-7

**Published:** 2017-06-13

**Authors:** Cristiano Rosafio, Serena Paioli, Cinzia Del Giovane, Valentina Cenciarelli, Nilla Viani, Paolo Bertolani, Lorenzo Iughetti

**Affiliations:** 1 Pediatric Unit, Post Graduate School of Pediatrics, University of Modena and Reggio Emilia, Viale del Pozzo, 71, 41124 Modena, Italy; 2 Hospital Pharmacy Unit, Post Graduate School of Hospital Pharmacy, University of Modena and Reggio Emilia, Viale del Pozzo, 71, 41124 Modena, Italy; 3 Department of Diagnostic, Clinical and Public Health Medicine, University of Modena and Reggio Emilia, Viale del Pozzo, 71, 41124 Modena, Italy; 4 Hospital Pharmacy Unit, Department of Pharmacy, University of Modena and Reggio Emilia, Viale del Pozzo, 71, 41124 Modena, Italy; 5 Pediatric Unit, Department of Medical and Surgical Sciences for Mothers, Children and Adults, University of Modena and Reggio Emilia, Viale del Pozzo, 71, 41124 Modena, Italy

**Keywords:** Medication related events, Medication-related event visits, Medication-related visit, Adverse drug events, Adverse drug reactions, Medication error, Emergency department

## Abstract

**Background:**

There are limited data on the characterization of medication-related visits (MRVs) to the emergency department (ED) in pediatric patients in Italy. We have estimated the frequency, severity, and classification of MRVs to the ED in pediatric patients.

**Methods:**

We retrospectively analyzed data for children seeking medical evaluation for a MRV over an 8 years period. A medication-related ED visit was identified by using a random pharmacist assessment, emergency physician assessment, and in case of conflicting events, by a third investigators random assessment.

**Results:**

In this study, regarding a single tertiary center in Italy, on a total of 147,643 patients from 0 to 14 years old, 497 medication-related visits were found, 54% of which occurred in children from 0 to 2 years of age. Severity was classified as mild in 21.6% of cases, moderate in 67.2% of cases, and severe in 11.2% of cases. The most common events were related to drug use without indication (51%), adverse drug reactions (30.3%), supratherapeutic dosage (13.2%) and improper drug selection (4.5%).

The medication classes most frequently implicated in an ADE were anti-infective drugs for systemic use (28.9%), central nervous system agents (22.3%) and respiratory system drugs (10.8%).

The most common symptom manifestations were dermatologic conditions (46.1%), general disorder and administration site conditions (29.7%) and gastrointestinal symptoms (16.0%).

**Conclusions:**

To our knowledge, this is the first study in Italy evaluating the epidemiologic characteristics of MRVs confirming a significant cause of healthcare contact resulting in ED visits and hospital admissions with associated resource utilization. Our results suggests further future prospective, large-sample sized, and multicenter research is necessary to better understand the impact of MRVs and to develop strategies to provide care plans and monitor patients to prevent medication-related visits.

**Trial registration:**

Not applicable.

## Background

Medication related events (MREs) are disadvantageous occurences, mostly preventable, related to use of a drug [1, 2].

It has been estimated that MREs are a significant problem in term of impact of consultations in Emergency Department (ED) and hospital admission in pediatric patients, contributing to overall pressures on health care system [3–5]. Previous studies focused on adverse drug events (ADEs) involved different methodologies and a spectrum of different inclusion criteria, ranged from studies of narrowly defined adverse drug reactions (ADRs) to more broadly defined medication-related events” [4, 6–44].

The Italian active pharmacovigilance project ‘Monitoring of the adverse effects in pediatric population’ (MEAP), aimed to assess pediatric ADRs, provided a valid strategy to identify previously unknown ADRs, reduce underreporting and increase awareness in pediatric clinical practice [45]. This project however is focused exclusively on pediatric ADRs, therefore little is known about pediatric ADEs [46–48] and no study has previously explored pediatric MREs, in Italy.

Thus there remains a significant knowledge gap in our understanding the magnitude of MREs in pediatric population. The purpose of this report is to explore retrospectively the phenomenon of MREs in pediatric patients that result in ED visit.

## Methods

We performed a retrospective cohort analysis reviewing all available electronic ED charts, collecting data from visits by children 0 to 14 years from 2007 to 2014 presenting to the ED of a single tertiary center, Azienda Ospedaliero-Universitaria Policlinico of Modena.

Independent investigators were full trained in predefined data-extraction criteria and the classification of Medication-Related Event visits (MRV) using a predefined approach [18, 39] Cases were randomly assigned. In case of ambiguous or conflicting events, a third investigators resolved the conflicts by discussing with the reviewers to achieve consensus [49].

Data were recorded anonymously on computerized abstraction forms (Microsoft Access 2011, Microsoft Corporation, Redmond, WA).

An ED visit was considered medication-related if the presentation was unequivocally related to the presenting chief concern and codified into 1 of 8 predefined categories, Table 1: ADR, drug interaction, improper drug selection, untreated indication, sub therapeutic dosage, supra-therapeutic dosage, non-adherence, and drug use without indication [50–53].

**Table 1:** Categorization of Drug Related Problems [49]

ADR	Any noxious, unintended, or undesired effect of a drug that occurs at doses used in humans for prophylaxis, diagnosis, or treatment. These will include all reactions when drugs are used at appropriate doses and may include abnormal laboratory values.
Untreated indication	Any noxious, unintended, or undesired effect resulting from the failure to treat a known indication
Improper drug selection	Any noxious, unintended, or undesired effect due to the use of a drug not optimal in the treatment of a confirmed indication
Subtherapeutic dosage	Anynoxious,unintended,orundesiredeffectcausedbyfailuretoreceive sufficient drug dosage or duration for a given indication or patient
Supratherapeutic dosage	Any noxious, unintended, or undesired effect caused by excessive drug dose or duration for a given indication or patient
Nonadherence	Any noxious, unintended, or undesired effect caused by failure to receive a drug as prescribed by a health care provider
Drug use without indication	Any noxious, unintended, or undesired effect caused by the use of a drug for which there is no clear indication
Drug interaction	Any noxious, unintended, or undesired effect caused by the coadministration of 2 or more drugs

Both Naranjo Scale and World Health Organization (WHO) algorithm was used to determine causality [54, 55].

We considered an adverse drug related event as present if the WHO algorithm was deemed “certain” or “probable” or the Naranjo Scale was deemed “definite” or “probable” [18].

MRV severity was ranked as fatal, severe (life threatening or resulting in permanent disability), moderate (laboratory abnormality or symptom requiring treatment/hospitalization or resulting in non-permanent disability) and mild (laboratory abnormality or symptom not requiring treatment) [14, 18]. Potential MRV was included in our study. A potential MRV was defined as incidents with potential for injury related to a drug [7]. If treatment was started in order to prevent medical consequences we considered it as a potential MRV. We decided to evaluate potential MRV on the assumption that the causes of potential MRV are similar to the causes of true MRV [7].

We excluded drugs used for intentional self-harm and event caused by illicit drugs, nicotine, ethanol, nutritional supplements, complementary and alternative medication and assault by poisoning. Furthermore, we excluded ED visits that were made by the same patient.

Narrative summaries, clinical testing, and physician’s diagnoses from MRVs reports were coded according to Medical Dictionary for regulatory Activities (MedDRA) terminology and System Organ Class (SOC) list [56].

Medication involved in each event was classified according to anatomical therapeutic classification code (ATC).

The study was performed on the basis of the rules of Ethical Committee.

Descriptive analysis (mean and standard deviation for continuous data, absolute frequency and percentage for categorical data) was performed with STATISTICA™ software (StatSoft Inc., Tulsa, OK, USA).

The primary outcome (Number of MRVs, Severity of True MRVs and Classification) of emergency department visits that were drug-related is reported as a percentage with 95% confidence interval (CI).

## Results

Over the 8-year period a total of 147.643 patients presented to the ED, with a mean (SD) of 18.455 (805.7) visits per year.

A total of 497 MRVs (0.34%, 95% CI 0.31% to 0.37%) were identified with the highest proportion of visits by children 0–2 years old who accounted for 54% of visits. Drug use without indication-related visit accounted for 257 cases (51%) followed by ADR-related visits 152 cases (30,3%), supratherapeutic dosage-related visit 66 cases (13.2%) and improper drug selection-related visit 22 cases (4.5%) Table 2.

**Table 2:** Demographic findings of patients (*n* = 497)

Characteristics			n(%, 95% CI)	
Age, mean (SD, CI 95%)			3.2(2.9, 95% CI 0.31% to 0.37%))	
age category				
0–2 y			269 (54)	
2–6 y			168 (33)	
6–14			60 (13)	
Gender				
girl			225 (45)	
Boy			272 (55)	
Classification of MRVs (*n* = 497)				
Drug Use Without Indication			257(51)	
ADR			152(30.3)	
Supratherapeutic Dosage			66(13.2)	
Improper Drug Selection			22(4.5)	
True MRVs			232 (46.5)	
ADR			152(65.5)	
Medication related-ADRs			87(37.5)	
Vaccination Related-ADRs			65(28)	
Drug Use Without Indication			46(19.8)	
Supratherapeutic Dosage			26(11.2)	
Improper Drug Selection			8(3.5)	
Potential MRVs			265 (53.5)	
Drug Use Without Indication			211 (79.6)	
Supratherapeutic Dosage			40(15.1)	
Improper Drug Selection			14(5.3)	
Degree of severity				
		True MRVs (*n* = 232)	Medication related-ADRs (*n* = 87)	VACCINES (*n* = 65)
	Mild	50 (21.6, 95% CI 7.5% to 16.0%)	16 (18.4)	12 (18.5)
	Moderate	156 (67.295% CI 60.7% to 73.2%)	66 (75.8)	52 (80)
	Severe	26 (11.2, 95% CI 16.4% to 27.4%)	5 (5.8)	1 (1.5)
Outcome				
	Hospitalization		34(6.8)	
	Death		-	

Potential MRVs was found in 265 cases (53.5%) while a true MRV in 232 cases (46.5%).

Among true MRVs the most common events were related to ADRs 152(65.5%), followed by drug use without indication 46(19.8%), supratherapeutic dosage 26(11.2%), and improper drug selection 8(3.5%) while among potential MRVs the most common events were related to drug use without indication 211(79.6%), followed by supratherapeutic dosage 40(15.1%) and improper drug selection 14(5.3%).

Medication-related ADRs accounted for 87 cases (37.5%) while Vaccination-related ADRs accounted for 65 cases (28%).

Severity classification was for True MRVs, mild in 50 cases (21.6%, 95% CI 16.4% to 27.4%), moderate in 156 cases (67.2%, 95% CI 60.7% to 73.2%), severe in 26 cases (11.2%, 95%CI 7.5% to 16.0%), for medication-related ADRs, severe in 5 cases (5.8%), moderate in 66 cases (75.8%), mild in 16 cases (18.4%) and for Vaccine-related ADRs mild in 12 cases(18.5%), moderate in 52 cases(80%) and severe in 1 case (1.5%).

The medication classes most frequently implicated in the total MRVs were anti-infective drugs for systemic use (28.9%, 95% CI 25.0% to 33.1%), central nervous system agents (22.3%, 95% CI 18.7% to 26.2%) and respiratory system drugs (10.8%, 95% CI 8.3% to 13.9%) Table 3.

**Table 3:** Medication Associated To MRVs

ATC classes								
	Total MRVs (*n* = 497) n(%)		True MRVs (*n* = 232) n(%)		Potential MRVs (*n* = 265) n(%)		Medication-related ADRs (*n* = 87) n(%)	
Antiinfectives for systemic use	144	(28.9)	129	(55.6)	15	(5.6)	52	(59.7)
Vaccines	65	(45.1)	65	(50.3)	-	-	-	-
Beta-Lactam Antibacterials	59	(40.9)	47	(36.4)	12	(80.0)	43	(82.6)
Macrolides, Lincosamides and Streptogramins	15	(10.4)	12	(9.3)	3	(20)	5	(9.6)
Nervous System	111	(22.3)	40^a^	(17.2)	71	(26.7)	7	(8.0)
Paracetamol	34	(30.6)	13	(32.5)	21	(29.5)	1	(14.2)
Psycholepticts	29	(26.1)	9	(22.5)	20	(28.1)	-	-
Antidepressants	17	(15.3)	7	(17.5)	10	(14.0)	-	-
Antiepileptics	14	(12.6)	5	(12.5)	9	(12.6)	3	(42.8)
Opioids	5	(4.5)	1	(2.5)	4	(5.6)	1	(14.2)
Respiratory System	54	(10.8)	14^b^	(6)	40	(15	6	(6.8)
Adrenergics, Inhalants	18	(33.3)	8	(57.1)	10	(25)	3	(50)
Antihistamines for Systemic Use	17	(31.4)	3	(21.4)	14	(35)	-	-
Cough and Cold Preparations	10	(18.5)	2	(14.2)	8	(20)	2	(33.3
Alimentary Tract and Metabolism	52	(10.4)	15^c^	(6.4)	37	(13.9	7	(8.0)
Drugs for Functional Gastrointestinal Disorders (domperidone, metoclopramide, cimetropium bromide)	15	(28.8)	4	(26.6)	11	(29.7)	2	(28.5)
Stomatological Preparations	10	(19.2)	1	(6.6)	9	(24.3)	-	-
Blood Glucose Lowering Drugs, Excl. Insulins	7	(13.4)	4	(26.6)	3	(8.1)	-	-
Vitamins	6	(11.5)	2	(13.3)	4	(10.8)	1	(14.2)
Drugs for Acid Related Disorders	4	(7.6)	-	-	4^11^	(36.3)	-	-
Antidiarrheals, Intestinal Antiinflammatory/Antiinfective Agents	4	(7.6)	1	(6.6)	3	(8.1)	1	(14.2)
Drugs for Constipation	3	(5.7)	1	(6.6)	2	(5.4)	1	(14.2)
Antiemetics and Antinauseants	2	(3.8)	2	(13.3)	-	-	2	(28.5)
Musculo-Skeletal System	36	(7.2)	14	(6)	22	(8.3)	11	(12.6)
NSAIDs (ketoprofen, ibuprofen)	31	(86.1)	14	(100)	17	(77.2)	11	(100)

^a^18(45) cases were drug use without indication-related event, 10(25) cases were supratherapeutic dosage-related event, 5(12.5) cases were improper drug selection-related event;

^b^5(35.7) cases were drug use without indication-related event, 2(14.2) cases were supratherapeutic dosage-related event, 1(7.1) cases were improper drug selection-related event;

^c^5(33.3) cases were drug use without indication-related event, 3(20) cases were supratherapeutic dosage-related event;

Among MRVs related to anti-infective for systemic use 45.1% were the result of a vaccine, 40.9% were the result of a beta-lactam antibacterial and 10.4% of a macrolides.

Central nervous system agents-related events were most often linked to use of paracetamol (30.6%), psycholepticts (26.1%), antidepressants (15.3%) and antiepileptic drugs (12.6%). The majority of MREs involving respiratory system agents were associated with adrenergic inhalants (33.3%) and antihistamines for systemic use (31.4%).

Anti-infective drugs for systemic use (55.6%) and Nervous system agents (17.2%) are the major medication classes involved in true MRVs with respectively Vaccines (50.3%), beta-lactam antibacterials (36.4%), paracetamol (32.5%) and psycoleptics (22.5%). The major categories of medications involved in medication-related ADRs included anti-infective for systemic use (59.7%) with an 82.6% of beta-lactam antibacterial involved while nervous system agents are the most common drug classes involved in MRVs not classifiable as an ADR. Table 4.

**Table 4:** Medication associated to MRVS not related to ADRs

ATC classes	True Drug use without indication *n* = 46 n(%)	Potential Drug use without indication *n* = 211 n(%)	True Supratherapeutic dosage *n* = 26 n(%)	Potential Supratherapeutic dosage *n* = 40 n(%)	True Improper drug selection *n* = 8 n(%)	Potential Improper drug selection *n* = 14 n(%)
Nervous System	18(39.1)	52(24.6)	10(38.4)	12(30)	5(62.5)	6(42.8)
Cardiac	5(10.8)	27(12.8)	-	-	1(12.5)	-
Respiratory System	5(10.8)	27(12.8)	2(7.7)	9(22.5)	1(12.5)	4(28.5)
Alimentary Tract and Metabolism	5(10.8)	24(11.3)	3(11.5)	9(22.5)	-	4(28.5)
Musculo-Skeletal System	3(6.5)	22(10.4)	-	-	-	-
Antiinfectives For Systemic Use	3(6.5)	6(2.8)	9(34.6)	9(22.5)	-	-
Genito Urinary System and Sex Hormones)	1(2.1)	22(10.4)	-	-	-	-

Dermatologic conditions were the most common true MRVs and Medication-Related ADRs manifestation, present respectively in 46.1% and in 88.5% of cases of true MRVs followed by general disorder and administration site conditions in 29.7% of cases and by gastrointestinal symptoms in 16.0% of cases of medication related ADRs. Table 5.

**Table 5:** Symptoms of pediatric MRVs

Symptoms								
	True MRVs n(%)		Medication related-ADRs n(%)		Vaccination Related-ADRs n(%)	Drug use without indication n(%)	Supratherapeutic dosage n(%)	Improper drug selection n(%)
Skin and subcutaneous tissue disorders	107	(46.1)	77	(88.5)	28 (43.0)	1(2.1)	1(3.8)	-
General disorders and administration site conditions	69	(29.7)	9	(10.3)	54 (83.0)	3(6.5)	3(11.5)	-
Gastrointestinal disorders	56	(24.1)	14	(16.0)	8 (12.3)	18(39.1)	14(53.8)	2(25)
Nervous system disorders	30	(12.9)	4	(4.6)	1 (1.5)	16(34.7)	4(15.3)	5(62.5)
Eye disorders	15	(6.4)	9	(10.3)	2 (3.0)	1(2.1)	1(3.8)	2(25)
Respiratory, thoracic and mediastinal disorders	15	(6.4)	4	(4.6)	6 (9.2)	-	4(15.3)	1(12.5)
Psychiatric disorders	12	(5.1)	2	(2.3)	4 (6.1)	5(10.8)	1(3.8)	-
Musculoskeletal and connective tissue disorders	7	(3.0)	1	(1.1)	3 (4.6)	-	2(7.6)	1(12.5)

General disorders and administration site conditions were the most common Vaccination-Related ADR manifestation, present in 83.0% of cases followed by dermatologic conditions in 43.0% of cases. Table 5.

In 41.5% of cases the type of vaccines was not reported, severity was classified mostly moderate (80% of cases) and type of reaction was manly systemic. Among the rest of available type of vaccines, Hexavalent (13.8%), DTaP-MMR(12%) and MMR(7.6%) were predominantly associated with an MRV with a mild-moderate severity classification. Table 6.

**Table 6:** Reactions reported for each type of vaccines (*n* = 65)

			Type of reaction		
TYPE n(%)			Systemic	Local	Systemic and Local
Not Reported	27	(41.54)	21	4	2
Hexavalent	9	(13.85)	7	2	
DTaP, MMR	8	(12.31)	1	5	2
MMR	5	(7.69)	2	1	2
MMR, MEN C	3	(4.62)	3		
DTaP, IPV	3	(4.62)		2	1
HPV	2	(3.08)	2		
PCV	2	(3.08)	1	1	
PNEUMOVAX 23	2	(3.08)			2
BCG	1	(1.54)		1	
Hepa B	1	(1.54)	1		
MEN C	1	(1.54)		1	
MMR, DTP, IPV	1	(1.54)		1	

*DTaP* difto-tetanus-pertussis vaccine, *IPV* inactivated antipolio vaccine, *Hepa B* Hepatitis B vaccine, *Hib* Haemophilus Influenzea type B vaccine, *MMR* measles, mumps, rubella vaccine, *PCV* pneumococcal eptavalente conjugate vaccine, *MEN C* meningococcal C conjugate vaccine, *PNEUMOVAX 23* pneumococcal vaccine polyvalent, *HEXAVALENT* DTaP/Hib/IPV/HepB vaccine, *BCG* bacille Calmette-Guérin (BCG) vaccine

## Discussion

Definition and classification of ADEs was not standardized across different studies so we decided to study MREs following the more comprehensive and reproducible definition of Hepler and Strand [50]. This taxonomy provides a more accurate and inclusive evaluation of drug-related event and a meaningful characterization of MRVs in ED. In our analysis MRVs accounted for 0.34% of the ED visits appearing similar to the range of 0.5% to 3.3% found in previous studies [32, 52, 57–60].

To our knowledge the extent of MRVs presenting to ED, exclusively among the pediatric population, has not been previously studied in Italy.

Comparisons with the other Italian studies is challenging since there are limited researches that have investigated pediatric ADEs presenting to ED. Capuano et al. reported only 3(0.41%) patients aged 0–19 years old presenting an ADE [46], Trifirò et al. reported 39(1.9%) patients aged 0–19 years old [48] and Raschetti et al. reported 10(3.8%) patients aged 0–20 years old [47]. Considering these studies, the sample size and the age range limit the possibilities for comparison.

More than two-thirds of true MRVs were identified as being moderate and mild as most commonly described in previous studies [32, 44, 52, 61, 62].

The rate of hospitalization (34 cases, 6.8%) was a slightly greater than reported in the literature (0.16–4.3%) [63]. This increase should be interpreted in the light of the need to a prolonged period of observation.

As previously reported [52, 58, 64, 65] drug use without indication related visits and ADRs are the most common MRVs. These most commonly occurred after intake of anti-infective for systemic use agents, nervous system drugs and respiratory system agents causing dermatologic manifestation followed by general disorders, administration site conditions and gastrointestinal manifestation.

Among medication related-ADRs dermatological manifestation after beta-lactam antibacterial use is the most common disorder which is consistent with the findings of the major national ADRs overview conducted in Italy from 2001 to 2012, collecting data from the Italian Pharmacovigilance Network database [45, 66].

Consistency of our data could be explained by a partial overlapping of the nature of pediatric population across the countries. This could probably be an echo of the scenery of disease prevalence and correlated medication use in children.

The results of research in adults could not be generalizable to children [67].

The European Regulation on Medicines for Pediatric use came into force on 26 January 2007 increased premarketing drug safety but efforts should take place at all levels to improve drug safety in a ‘real-life’ setting [66].

Kozer et al. [68] proposed some strategies that have the potential to reduce pediatric drug-related problems focusing on a ‘system approach’ view in which a medical error is a system error. PROTECT initiative [69], developed by The Centers for Disease Control and Prevention, delineated the key priorities for early action aimed to reduce medication errors.

There are several limitations in our study. First, the retrospective design of this study could underestimate the true incidence of MRVs. We consider the assessment of an experienced pharmacist and an independent physician both trained in recognition and resolution of MRVs to increase likelihood that all medication-related causes of an ED visit were identified and minimize potential misclassification.

Although we used an independent adjudication, process bias may have occurred in the categorization of the case summaries. Furthermore, retrospective analysis of data is limited by the impossibility to obtain additional information on the type of drug reaction.

Second, physicians may not have recognized a symptom as related to a medication; patient’s report could have been incomplete or presenting with multiple problems. So it is possible that some cases were not classified as a MRV.

Third, conditions with significant impact like medication nonadherence and the related increased health care use in children and adolescent who have chronic medical condition were not evaluated [70].

Finally given the retrospective nature of the study and the involvement of one single hospital, our results are not necessarily generalizable to the pediatric ED setting.

## Conclusion

MRVs are common medical complications among children in our setting and a challenge for the health care system. Our results are consistent with those from the current literature. Future prospective, large-sample sized, and multicenter research should focus in different settings on this topic to better understand the impact of MRV and the real effect of programmed preventive actions.

## Abbreviations

ADEs: Adverse drug events
ADRs: Adverse drug reactions
ATC: Anatomical therapeutic classification code
ED: Emergency department
MEAP: Monitoring of the adverse effects in pediatric population
MedDRA: Medical Dictionary for regulatory activities terminology
MREs: Medication related events
MRV: Medication-Related Event visit
SOC: System Organ Class
